# Editorial: Chronic Inflammation and Related Diseases: From Mechanisms to Therapies

**DOI:** 10.3390/ijms241310460

**Published:** 2023-06-21

**Authors:** Suk-Yun Kang, Yeonhee Ryu

**Affiliations:** KM Science Research Division, Korea Institute of Oriental Medicine, Daejeon 34054, Republic of Korea; sy8974@kiom.re.kr

The purpose of this Special Issue is to identify the exact mechanism underlying inflammation to direct more effective strategies for inflammation management and to provide basic data for the development of anti-inflammatory and analgesic treatment methods for patients with inflammatory pain. Inflammation and pain are complex processes that play crucial roles in the body’s response to injury, infection, and tissue damage. Inflammation serves as a critical defense mechanism against harmful stimuli. It is activated in response to tissue damage or infection, and aids in initiating the healing process. Pain is an unpleasant sensory and emotional experience associated with tissue damage or potential tissue damage that alerts the body to possible danger and is a protective mechanism that helps prevent further injury. However, the dysregulation of these processes can lead to chronic pain and inflammatory diseases. Therefore, it is crucial to understand the molecular mechanisms that regulate inflammation and pain.

In this Special Issue, researchers in the fields of inflammation and pain investigate the precise molecular mechanisms underlying these processes. As a result, the publications include 10 experimental papers focusing on inflammation and pain, along with 3 review papers. The papers published in this Special Issue describe a range of experiments that explore different types of inflammation, including colitis, neuropathic pain, obesity, intestinal disease, chronic kidney disease, memory dysfunction, sepsis, and asthma. These studies also discuss the various mechanisms associated with inflammation and immunity, providing a comprehensive understanding of the subject [1,2,3,4,5,6,7,8]. Studies investigating the mechanism of pro-inflammatory cytokines, such as interleukin-6 (IL-6) and tumor necrosis factor-alpha (TNF-α), have gained attention, and cover a wide range of topics, including the role of transient receptor potential (TRP) channels, glial cells, mitogen-activated protein kinase, ion channels, and signaling pathways in inflammation and pain.

The ongoing coronavirus disease (COVID)-19 pandemic has increased attention on the importance of immunity in protecting individuals from infectious diseases. The COVID-19 epidemic killed countless people and led to a global crisis. Consequently, researchers studying inflammation and immunity are conducting extensive research to better understand the immune response and develop effective treatments and vaccines in response to the global health crisis. One of the key findings is the phenomenon termed the “cytokine storm,” which is characterized by the sudden and intense production of inflammatory cytokines, such as IL-6 and TNF-α, and is observed in various infectious and immune-mediated conditions. This condition leads to strong and rapid activation of the immune system [9].

According to Chen et al., patients with severe acute respiratory syndrome coronavirus 2 (SARS-CoV-2) infection exhibit elevated inflammatory cytokine levels. Furthermore, the study emphasized the notable impact on T lymphocytes, which could potentially lead to a decrease in the production, and thereby the levels, of interferon gamma (IFN-γ) [10]. In another study, researchers analyzed the immune cell response to SARS-CoV-2 and found that more than 70% of recovered patients exhibited the presence of CD4+ and CD8+ T cells. Interestingly, the T cell response was not restricted to the Spike protein, but also included other proteins such as M, N, and various other open reading frames (ORFs). Furthermore, it was also observed that the T cell response to SARS-CoV-2 epitopes could be detected in individuals who had not been directly exposed to the virus [11]. One study published in this Special Issue discusses the crucial role of Forkhead box O transcription factors (FoxOs) in maintaining normal cellular physiology. The researchers emphasize that FoxOs regulate the development and maturation of T and B lymphocytes and secretion of inflammatory cytokines. Additionally, it highlights the important functions of FoxO in these cellular processes [12].

This Special Issue also presents evidence supporting the application of alternative and complementary medicines for the management of inflammation and pain. The research presented highlights the potential of alternative therapeutic approaches to control these conditions, providing additional options beyond conventional treatments.

Kim et al. reported that the administration of diosgenin, a botanical steroidal saponin, to animals with neuropathic pain suppressed the transient receptor potential (TRP) V1 in the dorsal root ganglia (DRG) and cytokines in the spinal cord, resulting in analgesic effects [2]. TRPV1 is widely expressed in the nervous system and is involved in the transmission and control of pain. It also plays a crucial role in mediating thermal hyperalgesia during inflammation. Another study showed that both TRPV1 and NMDA receptors in the spinal cord play a role in transmitting inflammatory pain and that TRPV1 inhibition could effectively alleviate inflammatory pain by modulating NMDA receptors [13]. Cannabidiol (CBD), isolated from the plant extract of *Cannabis sativa*, which has a long history of medicinal use, is a well-known compound with reported analgesic and anti-inflammatory effects in various animal models of neuropathic pain, inflammatory pain, and arthritis. CBD inhibits lipopolysaccharide (LPS)-induced nociception by suppressing the expression of Toll-like receptor 4 (TLR4) through the in vivo cannabinoid system [14]. Further, the combination of CBD and tetrahydrocannabivarin lead to a more substantial improvement in the pain behavioral response compared to single administration in chemotherapy-induced neuropathic pain. This combination also regulated the expression of several pain-related proteins, including p-AMPK, SIRT1, PI3K, p-AKT, and p-P38 MAPK, in the DRG [15]. In a study published in 2019, the researchers used a mouse model to investigate the immunomodulatory and anti-inflammatory effects of curcumin, a turmeric compound. The study demonstrated that curcumin decreased lung inflammation in asthmatic mice by inhibiting the production of inflammatory cytokines and enhancing the expression of aquaporins [16]. Experiments to stimulate acupuncture points are also being actively researched as alternative and complementary approaches for controlling inflammation and pain. Bee venom (BV) therapy, which involves subcutaneous injection of diluted bee venom into acupuncture points, is a clinical practice used in Oriental medicine to alleviate pain. In animal experiments, bee venom therapy activates the descending ceruleospinal noradrenergic pathway and spinal cord alpha-2 adrenergic receptor [17]. In addition, repeated treatment with BV can inhibit the activity of spinal cord glial cells in animals with spinal cord injuries, resulting in analgesic and anti-inflammatory effects [18]. Electrical stimulation of acupuncture points suppress the expression of the spinal NMDA receptor NR2B subunit in chemotherapy-induced peripheral neuropathic pain, thereby reducing pain behavior and pain-related ultrasound vocalizations [19]. Collectively, these studies suggest that various natural products and/or alternative and complementary medicines, such as bee acupuncture or electrical stimulation, have the potential to play considerable roles in controlling inflammation and managing pain.

In conclusion, the research papers published in this Special Issue provide a comprehensive overview of the latest advancements in the understanding of the mechanisms underlying inflammation and pain control through animal experiments. Furthermore, they highlighted the potential of identifying new therapeutic targets for the effective treatment of inflammatory diseases and chronic pain. We hope that this Special Issue will direct further scientific inquiries into these important areas and contribute to the development of more effective treatments for patients with inflammation and pain.
